# Supplementary material for: Genetic Variants Underlying Plasticity in Natural Populations of Spadefoot Toads: Environmental Assessment versus Phenotypic Response
Source: Genes (Basel). 2024 May 11;15(5):611. doi: 10.3390/genes15050611 (PMC11121243; doi:10.3390/genes15050611)
Supplement: Supplementary file 1 [file genes-15-00611-s001.zip › genes-2982972-supplementary.pdf]

Supplementary Information for:

Genetic variants underlying plasticity in natural populations of spadefoot toads: Environmental assessment versus phenotypic response

Andrew J. Isdaner, Nicholas A. Levis, Ian M. Ehrenreich and David W. Pfennig

Supplementary Data S1: FaST-LMM GWAS scan results for all SNPs in the: 1) environmental assessment, 2) morph production across both ponds, 3) morph production in PO2-N, and 4) morph production in Horseshoe Pond.

Supplementary Data S2: Genes and transcripts found within a 200 Kb window of each QTL, including top BLAST hit genes from humans, *Xenopus tropicalis*, and *Nanorana parkeri*.

Table S1: Cross reference of *S. bombifrons* genome chromosome names and *S. multiplicata* pseudogenome assembly chromosome names.

***S. multiplicata* chromosome**	***S. bombifrons* chromosome**
Chr1	NC_017087
Chr2	NC_017088
Chr3	NC_017089
Chr4	NC_017090
Chr5	NC_017091
Chr6	NC_017092
Chr7	NC_017093
Chr8	NC_017094
Chr9	NC_017095
Chr10	NC_017096
Chr11	NC_017097
Chr12	NC_017098
Chr13	NC_017099

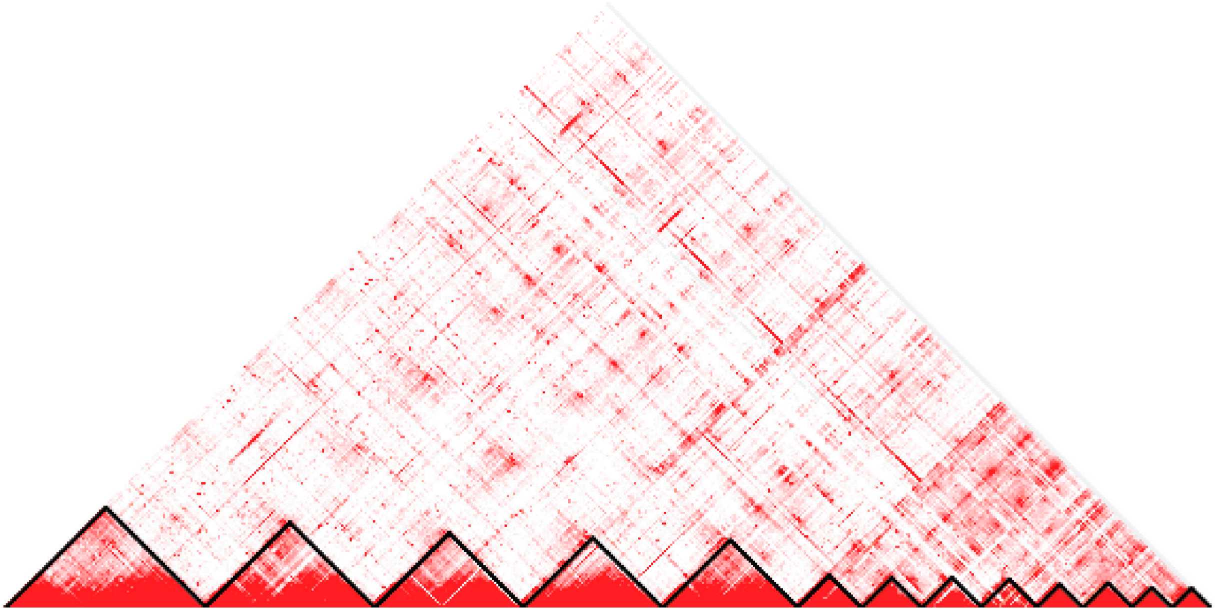

Figure S1: Heatmap of Hi-C contacts along the length of the *S. multiplicata* pseudogenome scaffolded on the *S. bombifrons* genome assembly. Black borders denote pseudogenome assembly chromosomes boundaries.

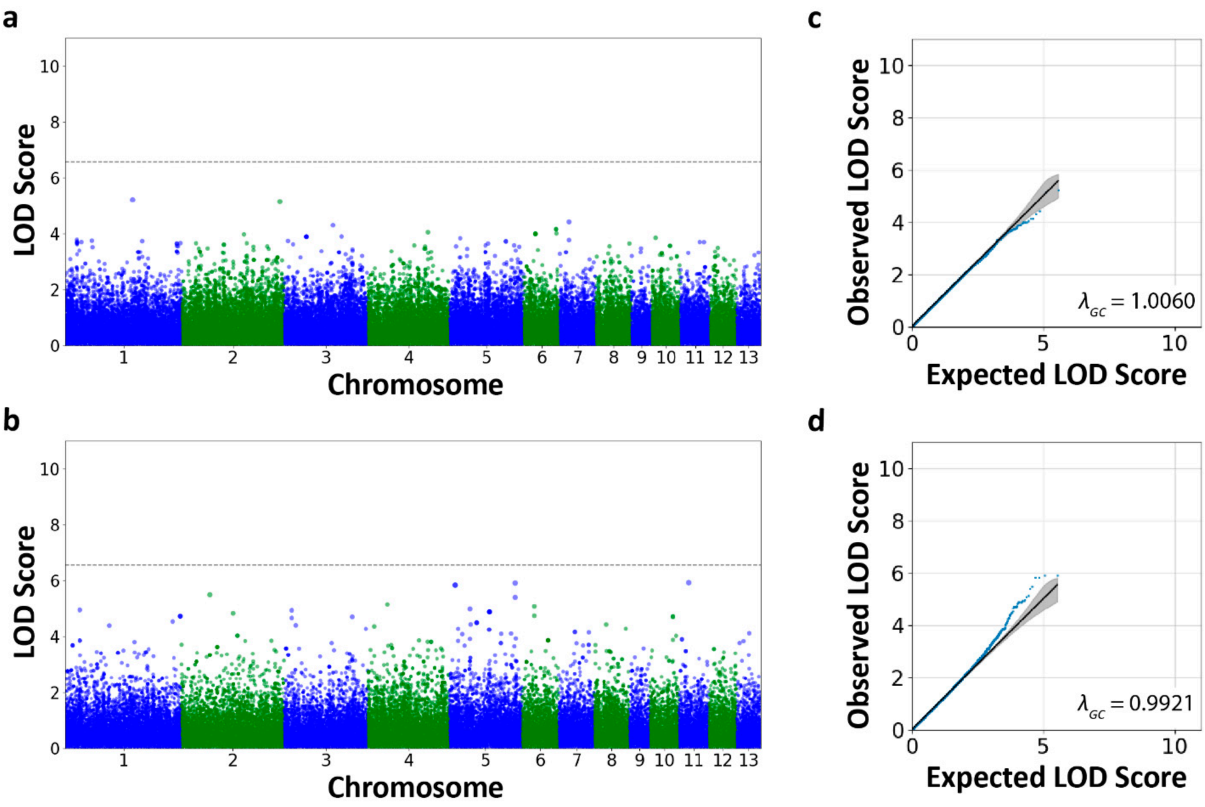

Figure S2: Manhattan plots of scans for QTL associated with a) morph production across both ponds (5% Bonferroni q-value = 2.696×10^{-7}) and b) morph production in PO2-N (5% Bonferroni q-value = 2.855×10^{-7}). Quantile-quantile plots of LOD scores of all loci for c) morph production across both ponds and d) morph production in PO2-N.

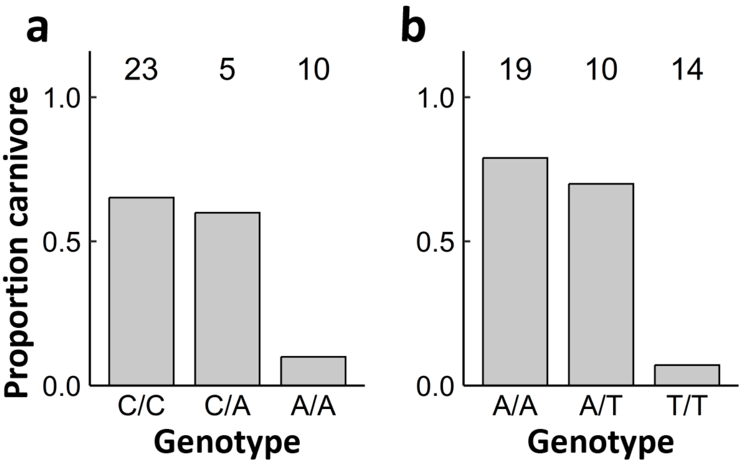

Figure S3: Individual association plots showing the proportion of carnivores produced in PO2-N with a given genotype at each locus that was found to be a statistically significant putative QTL in both the Horseshoe morph production scan and the environmental assessment scan. Note that these loci are *not* significant in the PO2-N morph production scan. Values above each bar represent sample size for each genotype. The QTL portrayed here are located at a) chromosome 3, position 19,873,503 and b) chromosome 5, position 90,293,461.

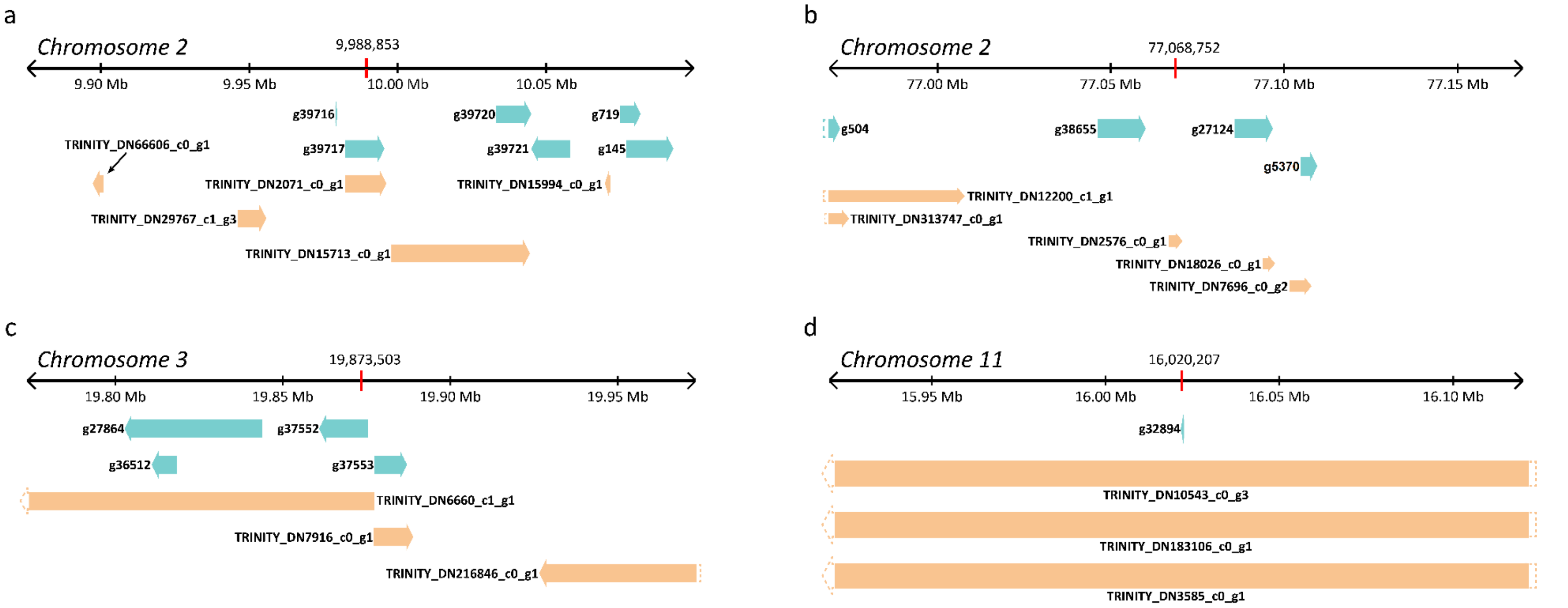

Figure S4. Genomic context of representative QTL, as discussed in the section 3.4, "Nearby Gene Analysis." The QTL is labelled with a red mark on the genomic axis, along with its precise genomic coordinates. Turquoise arrows represent gene annotations, and orange arrows represent transcript annotations. All annotated genes fall, at least in part, within a 200Kb window centered around each QTL. Transcripts shorter than 1,000 bp were excluded from this figure for visual clarity. The QTL depicted are respectively associated with a) morph production in Horseshoe, b) environmental assessment, c) both morph production in Horseshoe and environmental assessment, and d) environmental assessment.
